# MicroRNA Signatures Predict Brain Amyloidosis and Neurodegeneration in Alzheimer’s Disease: Insights from [^18^F] AV45 and FDG PET Imaging

**DOI:** 10.1002/brb3.70572

**Published:** 2025-05-26

**Authors:** Parsa saberian, Afra Darvishi, Delnia khezragha, Moojan Forouzandegan, Mohammad‐Erfan Farhadieh, Shaghayegh Taghizadeh Khorshidi, Mohamad Hatami Nejad, Sara Sedighi, Reza Barati, Seyed Ahmad Reza Safavi, Mohammad Sadeghi, David Gulisashvili, Mahsa Mayeli, Shayan Shakeri

**Affiliations:** ^1^ Student Research Committee, Faculty of Medicine Hormozgan University of Medical Sciences Bandar Abbas Iran; ^2^ Student Research Committee Tabriz University of Medical Sciences Tabriz Iran; ^3^ Faculty of Science University of Tabriz Tabriz Iran; ^4^ Students' Scientific Research Center Tehran University of Medical Sciences Tehran Iran; ^5^ Department of Cell and Molecular Biology and Microbiology, Faculty of Biological Sciences and Technology University of Isfahan Isfahan Iran; ^6^ Institute for Cognitive Science Studies Pardis Iran; ^7^ Department of Psychology, Faculty of Psychology and Educational Sciences University of Tehran Tehran Iran; ^8^ School of Medicine Iran University of Medical Sciences Tehran Iran; ^9^ Poursina Clinical Research Development Unit Guilan University of Medical Sciences Rasht Iran; ^10^ School of Medicine Tehran University of Medical Sciences Tehran Iran; ^11^ School of Rehabilitation Shiraz University of Medical Sciences Shiraz Iran; ^12^ Department of Diagnostic Radiology & Nuclear Medicine University of Maryland School of Medicine Baltimore Maryland USA; ^13^ Department of Medical Genetics, School of Medicine Shiraz University of Medical Sciences Shiraz Iran

**Keywords:** APOE ε4, Alzheimer's disease, beta amyloid, biomarker, cerebrospinal fluid, FDG‐PET, miRNA, neurodegeneration

## Abstract

**Purpose:**

Alzheimer's disease (AD) is a neurodegenerative disease primarily manifesting with cognitive decline. This study aimed to investigate the alterations in microRNAs in patients across AD continuum as potential biomarkers.

**Method:**

Data were extracted from the Alzheimer's Disease Neuroimaging Initiative (ADNI) database, including microRNA levels in the serum and cerebrospinal fluid (CSF) of patients across AD continuum. We analyzed the associations between microRNA levels and previously known AD biomarkers, such as amyloid beta (Aβ) accumulations in the brain and glucose reuptake values using positron emission tomography ([^18^F]AV45 and FDG PET, respectively).

**Findings:**

The study found a significant positive correlation between CSF levels of miR‐210‐3p and Aβ accumulations in the brain (B = 4.69). Conversely, miR‐223‐3p levels were significantly lower in APOE‐ε4 carriers. Significant negative correlations were also observed between glucose reuptake and several miRNAs in the AD group. Specifically, plasma levels of let‐7g‐5p, mir‐423‐5p, and mir‐660‐5p were negatively associated with glucose reuptake in the brain.

**Conclusion:**

Elevated levels of miR‐210‐3p correlate with Aβ accumulation, supporting previous findings of increased levels of certain microRNAs in patients with MCI and AD. Our findings highlight the potential of microRNAs as biomarkers of AD.

## Graphical abstract

This study leverages data from the Alzheimer's Disease Neuroimaging Initiative (ADNI) to examine differential microRNA expression in patients with MCI or mild AD. This graphical abstract illustrates key findings, including a significant positive correlation between CSF levels of miR‐210‐3p and Aβ accumulation (B = 4.69). Negative correlations were identified between‐ several microRNAs (let‐7g‐5p, miR‐423‐5p, and miR‐660‐5p) and glucose reuptake as measured by FDG‐PET. These results suggest that specific microRNAs may serve as potential biomarkers for AD progression.

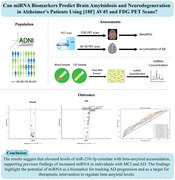

## 1 Introduction

Alzheimer's disease (AD) is a neurodegenerative disease with a hallmark of memory impairment. It is the most prevalent form of dementia in people older than 65. In fact, 6.5 million Americans were estimated to be living with AD in 2022. AD imposes a significant burden on public health, impacting cognitive function and quality of life for individuals and their caregivers. It also presents a substantial economic burden, with the annual cost of care in the United States surpassing $321 billion in 2022 and expected to increase (Akin et al. 2018). The prominent symptoms of AD include memory decline, language difficulties, and disorientation (Bature et al. 2017).

The exact pathogenesis of AD is still unclear, and no definite cure has been found. Evidence suggests that abnormal beta‐amyloid (Aβ) protein aggregation plays a role in the pathophysiology of AD. The Aβ precursor protein is a transmembrane protein expressed in many different cells, which can be cleaved in two pathways in the nervous system. If it is first cleaved by beta‐secretase, the pathway results in the production of Aβ. It is primarily a physiologic process, but the overactivity of this pathway causes Aβ to lose its solubility, leading to the formation of plaques (O'brien and Wong 2011). Plaque formation and amyloidosis have significant consequences in various diseases, particularly neurodegenerative disorders like AD. The accumulation of Aβ plaques in the brain disrupts normal neuronal function and leads to detrimental effects on cognition and memory (Sperling et al. 2009).

Categorization of AD biomarkers includes imaging and clinical diagnostic markers. Diagnostic imaging markers like positron emission tomography (PET) have good diagnostic accuracy in diagnosing AD and cognitive decline (Li et al. 2022). 18F‐fluorodeoxyglucose ([^18^F]‐FDG) PET, which represents the metabolism of glucose, can detect reductions in glucose reuptake in the brain, thus pointing to neurodegeneration in AD. [^18^F]‐FDG PET showed better results in predicting the conversion of mild cognitive impairment (MCI) to AD compared to single‐photon emission computerized tomography (SPECT) and magnetic resonance imaging (MRI) (Chételat et al. 2020, Laforce et al. 2018, Meza‐Sosa et al. 2012). [^18^F]‐FDG PET is reported to have higher specificity and short‐term predictive value than amyloid PET (Clerx et al. 2013). Some studies suggest that amyloid PET could be more useful in neurodegeneration assessment, and using combined (Chételat et al. 2020) tracers has a better performance than single tracer (Vanhoutte et al. 2021).

MicroRNAs (miRNAs) are a class of endogenous, single‐stranded, non‐coding RNAs, comprising 18–22 nucleotides. MiRNAs regulate post‐transcriptional expression via binding to target miRNAs. Consequently, alterations in their levels can be a critical factor affecting cellular processes and human disease. While most miRNAs are inside cells, a significant amount is released by cells into extracellular spaces (e.g., blood and cerebrospinal fluid (CSF)) under physiological and pathological conditions in the form of circulating miRNAs (Vergallo et al. 2021, Lee et al. 2020).

Several studies have investigated the role of miRNAs as potential biomarkers in MCI and AD. For instance, a study examined miRNA expression in the brains of AD patients and identified several dysregulated miRNAs, including miR‐29a, miR‐128a, and miR‐9. These miRNAs were associated with processes involved in AD pathogenesis, such as neuronal survival, amyloid precursor protein processing, and tau phosphorylation (Hébert et al. 2008). Another study by Hébert et al. explored miRNA expression patterns in the brains of individuals with AD and identified miR‐29, miR‐9, and miR‐132 as potential biomarkers. These miRNAs were associated with synaptic plasticity, neuronal survival, and inflammation, which are critical processes in AD progression (Hébert et al. 2008). Furthermore, a study investigated the peripheral blood miRNA expression profiles of AD patients and identified miR‐107, miR‐29a, and miR‐15b as potential diagnostic markers. These miRNAs were found to be associated with processes related to amyloidogenesis, oxidative stress, and neuroinflammation (Geekiyanage and Chan 2011).

MiRNA profiles may vary depending on the specific brain regions, sample types (brain tissue or peripheral blood), and disease stages analyzed in different studies. However, the dysregulation of miRNAs such as miR‐29, miR‐9, miR‐132, miR‐106b, miR‐520d, miR‐107, and miR‐15b has been consistently observed in various studies, suggesting their potential as significant miRNA alterations associated with AD (Lee et al. 2020). miRNA‐15b* time interaction has a positive effect on local uptake of [^18^F]‐FDG PET, placed in the left hippocampus, and a negative association exists between the miRNA‐125b plasma levels and glucose uptake in several regions (Vergallo et al. 2021).

Given that amyloidosis and glucose hypometabolism due to neurodegeneration are recognized as major markers of AD, we sought to evaluate the association between miRNAs and these factors, ultimately determining whether miRNAs can serve as potential diagnostic markers for AD. Additionally, we explored plasma and CSF changes in these miRNAs in patients with MCI and AD.

## 2 Methods

### 2.1 Participants

Data used to prepare this article were obtained from the Alzheimer's Disease Neuroimaging Initiative (ADNI) database (adni.loni.usc.edu). We included 160 participants (AD vs. healthy controls) who donated plasma and CSF collected under fasting conditions on the same day, for a total of 320 participant samples.

### 2.2 Sample Collection

The samples were analyzed using miRNA array cards(Lusardi et al. 2017, Sandau et al. 2020, Sandau et al. 2022, Sandau et al. 2020, Saugstad et al. 2017, Wiedrick et al. 2019). Total RNA was isolated from each biofluid and measured via qPCR using a custom TaqMan Low‐Density Array (TLDA) card in a 384‐well format based on TaqMan TLDA cards. Each card included 64 miRNA probes (192 total probes), and two participant samples were run on each card (384 wells total). The biofluids were processed for RNA isolation in November and December 2022 and then processed for qPCR in January and February 2023. Subsequent analysis included data quality measurement, normalizing, calibrating, and comparing relative quantities (Saugstad et al. 2017).

### 2.3 RNA Isolation

Each RNA isolation batch had 24 samples, including 12 donor‐ and date‐matched CSF and plasma samples. The samples were grouped together to ensure that individual donor samples were kept together, hence preventing any potential impact resulting from the RNA isolation batch. The biofluid samples were frozen on ice, and total RNA was extracted from 250 µL of CSF and 250 µL of plasma using the miRNA Purification kit (Norgen, Thorold, ON) according to the manufacturer's instructions. After adding the lysis buffer to the sample, a synthetic cel‐miR‐39‐3p spike‐in (Thermo Fisher, Waltham, MA) was introduced at a concentration of 3 picomolar (pM). CSF and plasma RNA were extracted using 30 µL and 50 µL of nuclease‐free water, respectively. The concentration of miRNA in each sample was quantified using the Qubit miRNA Assay kit (Thermo Fisher) and measured using a Qubit 4.0 fluorometer (Thermo Fisher).

### 2.4 miRNA Quality Control and Normalization

To ensure data reliability and minimize technical variability, a rigorous quality control (QC) and normalization protocol were applied to all miRNA expression data. Raw Cq values were examined to identify failed amplifications and outlier replicates. Probes that did not cross the detection threshold or showed inconsistent amplification across technical replicates were excluded from further analysis. Quality‐filtered data were then normalized using the global mean normalization method, which is considered robust for TaqMan Low‐Density Array (TLDA) platforms. In this approach, the average Cq of all detectable miRNAs within each sample was calculated, and the ΔCq for each miRNA was determined by subtracting the sample‐specific mean Cq from the raw Cq value. This method reduces sample‐to‐sample variation and enables comparison between CSF and plasma profiles. Additionally, the synthetic spike‐in cel‐miR‐39‐3p was used across all samples as an internal control to monitor RNA extraction efficiency and reverse transcription quality.

### 2.5 [^18^F]AV‐45 PET Scan

The imaging data from the ADNI dataset underwent a standardized preparation workflow. For precise information about imaging data acquisition, please refer to http://adni.loni.usc.edu/. Amyloid PET was performed using the florbetapir tracer (Chételat et al. 2020). The scan was conducted between 50 and 70 minutes following the injection. The generated images were subsequently averaged, aligned in space, interpolated to a standard voxel size, and smoothed. This procedure was executed to uniform the resolution of 8 mm whole width at half maximum (Jack et al. 2015).

### 2.6 FDG PET Scan

MetaROIs (regions of interest) were derived from the data for subjects’ FDG scans at each timepoint to compute intensity values. The FDG scans included samples of AD, MCI, and normal participants. MetaROIs were extracted from the most frequent regions implicated in AD and MCI. The processed format of PET data was then normalized in SPM to the MNI PET template.

### 2.7 Statistical Analysis

Continuous and categorical variables are shown as mean (SD) or median (interquartile range) and frequency (percentage), respectively. In the association between imaging modalities and the levels of miRNAs, multiple linear regression was utilized, adjusted for sex, age, education level, and APOE‐ε4 carrier status. Q values were determined through multiple independent t‐tests (Welch's t‐test) with false discovery rate (FDR) correction to account for multiple comparisons, and ROC curve analysis was performed to assess the diagnostic accuracy of the miRNA levels in predicting outcomes. miRNA‐gene network analysis was conducted to explore the associations between miRNAs and their target genes. Pathway analysis was carried out to identify the biological pathways enriched with the target genes of differentially expressed miRNAs.

## 3 Results

**FIGURE 1:**
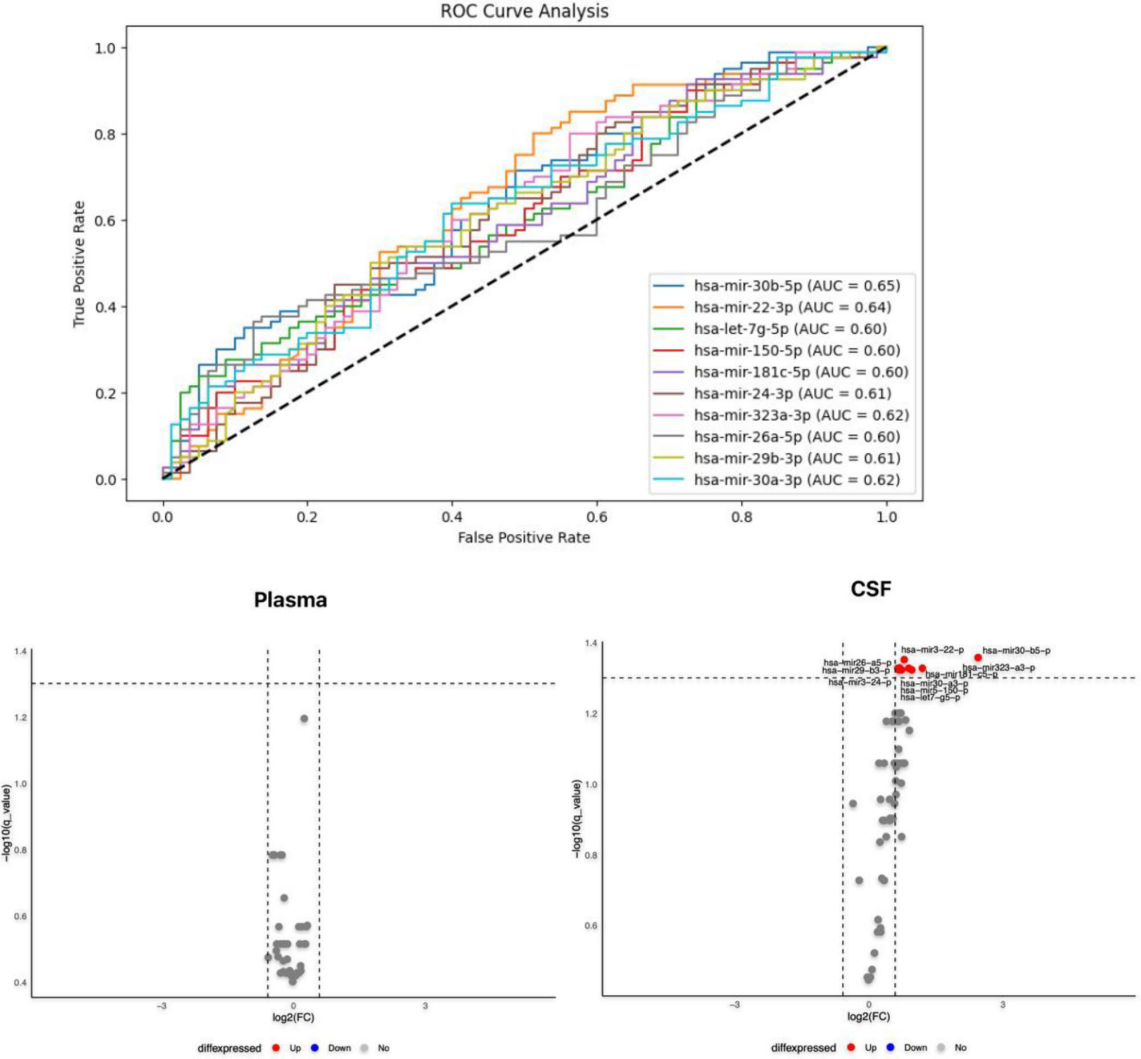
ROC curve analysis assessed miRNA levels' diagnostic accuracy in predicting outcomes.

Participants’ characteristics are summarized in Table 1. Following normalization and quality control checks, 62 unique miRNAs were examined in the CSF and plasma samples of the AD and HC groups. The plasma levels of none of the analyzed miRNAs were significantly different between the two groups after controlling for multiple comparisons (FDR < 0.05). We found significantly elevated levels of 10 miRNAs in the CSF in the AD group, including miR‐30b‐5p (p = 0.001), miR‐22‐3p (p = 0.001), miR‐26a‐5p (p = 0.036), miR‐323a‐3p (p = 0.008), miR‐29b‐3p (p = 0.013), miR‐181c‐5p (p = 0.022), miR‐30a‐3p (p = 0.008), miR‐24‐3p (p = 0.012), miR‐150‐5p (p = 0.027), and let‐7g‐5p (p = 0.022). These differences remained significant after FDR correction, and the diagnostic performance of each is illustrated in Figure 1 and Table 2.

**TABLE 1:** Demographic characteristics among study groups.

Characteristic	AD, N = 80*^1^*	CN, N = 80^1^	p‐value^2^
Age	75	74	0.3
Sex			0.2
Female	31 / 80 (39%)	39 / 80 (49%)	
Male	49 / 80 (61%)	41 / 80 (51%)	
Education	15.30 (2.47)	16.45 (2.60)	0.005
Number of APOE‐ε4 alleles			<0.001
0	25 / 80 (31%)	57 / 80 (71%)	
1	40 / 80 (50%)	21 / 80 (26%)	
2	15 / 80 (19%)	2 / 80 (2.5%)	
ABETA	718 (337)	1,243 (443)	<0.001
TAU	389 (153)	233 (86)	<0.001
MMSE	23.19 (1.86)	28.73 (1.42)	<0.001
CDR‐SB	4.71 (1.83)	0.03 (0.11)	<0.001

^1^ Mean (SD); n / N (%).

^2^ Welch Two Sample t‐test; Pearson's Chi‐squared test.

Abbreviations: CDRSB, clinical dementia rating scale sum of boxes; MMSE, mini‐mental state examination.

**TABLE 2:** Diagnostic potential of differentially expressed miRNAs in CSF samples of AD patients.

miRNA	AUC	p‐value	95% confidence interval
mir‐30b‐5p	0.65	0.001	0.56‐0.73
mir‐22‐3p	0.64	0.001	0.56‐0.72
mir‐7g‐5p	0.60	0.022	0.52‐0.69
mir‐150‐5p	0.60	0.027	0.52‐0.69
mir‐181c‐5p	0.60	0.022	0.52‐0.69
mir‐24‐3p	0.61	0.012	0.52‐0.70
mir‐323a‐3p	0.62	0.008	0.53‐0.71
mir‐26a‐5p	0.60	0.036	0.50‐0.68
mir‐29b‐3p	0.61	0.013	0.53‐0.70
mir‐30a‐3p	0.62	0.008	0.53‐0.71

Abbreviation: AD, Alzheimer’s disease; AUC, area under the curve; CSF, cerebrospinal fluid; miRNA, microRNA.

Multiple regression analyses in the AD and CN groups modeled the relationships between the miRNAs with Aβ load and FDG metabolism while adjusting for sex, age, education level, and APOE‐ε4 status as covariates. Table 3 reports the miRNAs that significantly correlated with either FDG‐PET or AV‐45 based on these models. Among the miRNAs in the CSF of AD patients, we observed a significant positive correlation between miR‐210‐3p levels and Aβ load (B = 4.690); conversely, there was a notable negative correlation between miR‐223‐3p levels and Aβ load (B = ‐2.192). In addition, we found significant positive correlations between the CSF levels of miR‐210‐3p and miR‐27b‐3p with FDG metabolism in AD patients (B = 4.834, 3.773). On the contrary, in the CN group, none of the mentioned correlations were found for these miRNAs in the CSF, while some different miRNAs were found to have correlations with Aβ load or FDG metabolism in this group. Among the miRNAs in the plasma, the levels of 4 miRNAs in the AD group showed significant negative associations with FDG metabolism: miR‐150‐5p, miR‐15a‐5p, mir‐323a‐3p, and miR‐92a‐39 (B = ‐2.059, ‐1.391, ‐3.681, ‐1.109.) However, in the plasma of the CN group, only miR‐192‐5p exhibited a significant negative correlation with FDG metabolism (B = ‐3.163).

**TABLE 3:** Significant correlations between miRNA levels with Aβ load (AV‐45) or FDG metabolism (FDG‐PET) based on the multiple regression models.

miRNA	variable	group	p‐value
**CSF**			
mir‐210‐3p	Aβ	AD	0.003
	FDG	AD	0.043^*^
mir‐223‐3p	Aβ	AD	0.007^*^
mir‐423‐5p	FDG	CN	0.025
let‐7b‐5p	FDG	CN	0.020
mir‐101‐3p	Aβ	CN	0.003
mir‐15a‐5p	Aβ	CN	< 0.001
mir‐19b‐3p	Aβ	CN	0.009^*^
mir‐23a‐3p	Aβ	CN	0.036^*^
mir‐26b‐5p	Aβ	CN	0.014^*^
mir‐374b‐5p	Aβ	CN	0.040^*^
mir‐153‐3p	FDG	CN	0.045^*^
mir‐190a‐5p	FDG	CN	0.020^*^
mir‐92a‐39	FDG	CN	0.025
mir‐92b‐3p	FDG	CN	0.020^*^
mir‐27b‐3p	FDG	AD	0.028^*^
mir‐100‐5p	FDG	CN	0.040^*^
mir‐150‐5p	FDG	CN	0.031^*^
mir‐186‐5p	FDG	CN	0.031^*^
mir‐200a‐3p	FDG	CN	0.018^*^
mir‐204‐5p	FDG	CN	0.020^*^
mir‐2110	FDG	CN	0.025^*^
mir‐376a‐3p	FDG	CN	0.036^*^
mir‐424‐5p	FDG	CN	0.042^*^
mir‐499a‐3p	FDG	CN	0.008^*^
mir‐502‐3p	FDG	CN	0.013^*^
mir‐647	FDG	CN	0.007^*^
**Plasma**			
mir‐15a‐5p	FDG	AD	0.034^*^
mir‐92a‐39	FDG	AD	0.040^*^
mir‐323a‐3p	FDG	AD	0.019^*^
mir‐150‐5p	FDG	AD	0.038^*^
mir‐192‐5p	FDG	CN	0.030^*^

* While some predictors show a potential relationship with Delta_Cq, the overall model's significance and predictive power are limited, indicating that these variables might not comprehensively explain changes in Delta_Cq for these miRNAs.

The regression models also revealed the associations of the covariates with the miRNA levels (Supplemental Table 1). We found significant correlations between the CSF levels of 3 miRNAs (let‐7g‐5p, mir‐423‐5p, and mir‐660‐5p) and APOE‐ε4 status in AD patients. The same tendency was observed for CSF levels of let‐7b‐5p and mir‐195‐5p in the CN group. Regarding the plasma levels, significantly negative associations of miR‐223‐3p and miR‐26a‐5p were found with APOE‐ε4 status in the CN group, while no such significant association was found among the miRNA levels of the AD group.

## 4 Discussion

We investigated the potential role of miRNAs as biomarkers of AD. miRNAs regulate the expression of genes in physiological and pathological cases, including neurological diseases (Meza‐Sosa et al. 2012). In AD, miRNAs such as miR‐210‐3p and miR‐223‐3p were correlated with Aβ burden and glucose reuptake in the brain. These miRNAs are known to be involved in hypoxia and inflammation, processes highly upregulated in AD pathology (Watts et al. 2018, Zhao et al. 2022). For instance, miR‐210‐3p has been associated with hypoxia‐inducible factors, which are upregulated in response to chronic hypoxic conditions in the AD brain. This may explain its correlation with both Aβ deposition and glucose metabolism in AD (Watts et al. 2018). Also, Siedlecki‐Wullich et al. illustrated that the relative level expression of miR‐210‐3p in plasma can discriminate between AD and healthy subjects, while we just find the association with Aβ load and FDG metabolism in CSF (Siedlecki‐Wullich et al. 2019). Conversely, miR‐423‐5p, which was correlated with FDG metabolism in the CN group, is known to directly inhibit the translation of mitochondrial cytochrome C mRNA (COX6A2), halting ATP production by the electron transport chain. This function may reflect a compensatory mechanism in the CN group, where aging neurons adapt to maintain energy homeostasis under metabolic stress. In contrast, its upregulation in AD is associated with disrupted mitochondrial function, emphasizing its dual role in aging and pathology (Noor Eddin et al. 2023). Interestingly, miR‐423‐5p was the only miRNA significantly associated with age, suggesting that its regulation may bridge aging‐related and disease‐specific processes. miR‐101‐3p, which was significantly correlated with Aβ load in the CN group, directly regulates the expression of amyloid precursor protein (APP) by binding to the 3′ untranslated region (UTR) of its mRNA transcript. This regulatory function prevents excessive APP expression and the activation of amyloidogenic pathways (Noor Eddin et al. 2023). The absence of this correlation in the AD group likely reflects a loss of this regulatory capacity, contributing to the overexpression of APP and subsequent Aβ accumulation in the disease state. miR‐15a‐5p, a potent negative regulator of ERK1, was correlated with Aβ load in the CN group. This miRNA is known to regulate tau phosphorylation pathways by modulating ERK1 activity. Reduced levels of miR‐15a‐5p in AD disrupt this regulation, resulting in increased ERK1‐mediated tau hyperphosphorylation and neurofibrillary pathology (Noor Eddin et al. 2023). This shift highlights the transition from protective mechanisms in aging to dysregulation in AD. let‐7b‐5p, a miRNA highly expressed in neurons and implicated in aging‐related pathways, was correlated with FDG metabolism in the CN group. This correlation may represent its role in maintaining neuronal energy metabolism during normal aging. However, in AD, the regulatory capacity of let‐7b‐5p may be overwhelmed by pathological processes, such as inflammation and synaptic dysfunction, that disrupt energy homeostasis (Wang et al. 2022). Our multiple linear regression analyses showed that the expression levels of most miRNAs were not significantly correlated with age, except for miR‐423‐5p. This finding suggests that the observed group‐specific miRNA correlations are not merely age‐related but rather reflect distinct biological processes in AD versus CN individuals. The significant associations of miRNAs with APOE‐ε4 status observed in our study may reflect the well‐established influence of APOE‐ε4 on lipid metabolism and inflammatory pathways, which are critical processes in AD pathology and aging. APOE‐ε4 disrupts lipid transport and homeostasis in the brain, leading to altered membrane composition, mitochondrial dysfunction, and increased oxidative stress, all of which are known to modulate miRNA expression. For instance, miR‐423‐5p, correlated with APOE‐ε4 status in the AD group, regulates mitochondrial energy metabolism and may represent a compensatory response to APOE‐ε4‐driven mitochondrial impairment. Similarly, let‐7b‐5p and let‐7g‐5p, which were associated with APOE‐ε4 status in both AD and CN groups, are regulators of inflammatory pathways and align with the pro‐inflammatory environment linked to APOE‐ε4. Furthermore, the negative associations of plasma miRNAs, such as miR‐223‐3p and miR‐26a‐5p, with APOE4 status in the CN group suggest that early dysregulation of inflammation and lipid metabolism might precede clinical symptoms. The findings in the CN group, particularly the significant associations of miRNAs with APOE‐ε4 status, provide valuable insights into preclinical and prodromal stages of AD. These miRNAs, including let‐7b‐5p and miR‐195‐5p, may reflect compensatory or protective mechanisms attempting to maintain neuronal and synaptic function in the face of APOE‐ε4‐related stressors. For example, let‐7b‐5p is involved in synaptic energy metabolism (Wang et al. 2022), and miR‐195‐5p regulates amyloid‐beta clearance and vascular integrity, processes that are crucial in mitigating early AD pathology (Noor Eddin et al. 2023). The observed plasma miRNA changes, such as those of miR‐223‐3p and miR‐26a‐5p, further highlight systemic early‐stage alterations in lipid and inflammatory pathways that could serve as biomarkers for identifying individuals at higher risk (Liu et al. 2022). Together, these findings underscore the potential of miRNAs as early biomarkers for APOE‐ε4‐mediated vulnerability and provide a foundation for exploring their role in targeted therapeutic interventions to delay or prevent progression to clinical AD. miRNAs are able to regulate several physiological pathways, reveal new molecular mechanisms, and suggest potential treatments, which makes studies in this field attractive (Awuson‐David et al. 2023). Research on miRNAs to date has identified the effects and cooperation of miRNAs in brain tissue, CSF, and peripheral blood, suggesting that miRNAs may play an important role as diagnostic biomarkers or as new therapeutic targets in AD (Sun et al. 2022). MiR‐30b affects synaptic integrity, and its upregulation in AD results in synaptic failure since it influences several target molecules such as EPHB2, SIRT1, and GluA2, which are downregulated as a result (Song et al. 2019). Also, miR‐30b is upregulated by proinflammatory cytokines, and therefore, activating NF‐κB, so inhibition of the NF‐κB pathway results in less expression of miR‐30b (Song et al. 2019). Since NF‐κB pathway activation is implicated in the pathogenesis of AD, it can be deduced that miR‐30b elevation leads to neuroinflammation, supporting our findings on the upregulation of miR‐30b in AD (Lukiw and Bazan 1998). Serum exosomal miR‐30b‐5p and miR‐22‐3p can be utilized as a model for predicting AD since they are notably dysregulated (Dong et al. 2021). Consistent with our results, the expression of miR‐30b‐5p, miR‐30a‐3p, miR‐24‐3p, miR‐22‐3p, and miR‐181c‐5p was significantly altered in the CSF of patients with AD. Studying plasma extracellular vesicles showed that specific miRNAs, including miR‐30b‐5p, are upregulated in AD patients compared to controls. Furthermore, the association between Aβ42 and miR‐30b‐5p was reported only in AD patients (Visconte et al. 2023). Studies on extracellular vesicles show that miR‐181a‐3p and miR‐24‐3p are both elevated in AD (Serpente et al. 2020). Expression of miR‐24‐3p is increased in AD, and a reverse relationship with the mini‐mental state examination score was observed, so it could serve as a diagnostic biomarker for AD (Liu et al. 2021). MiR‐22‐3p, a factor that plays an important role in brain development in some stages (less than 1 year or more than 3 years old group), is recognized to be altered in AD (Prieto‐Fernández et al. 2020). Increased levels of miR‐22‐3p in the hippocampus are shown to improve cognition in mice, decrease Aβ deposition, and amend cellular apoptosis. Thus, it rectifies AD by affecting Sox9, using NF‐κB signaling (Xia et al. 2022).

Multimodal neuroimaging techniques have been employed to improve early diagnosis and understand the pathological mechanisms underlying AD. FDG‐PET is a neuroimaging technique used to detect changes in glucose metabolism in the brain, and in AD, there are specific patterns of glucose metabolism alterations that FDG‐PET can identify (Akin et al. 2018, Martineau et al. 2021, Minamimoto et al. 2020). In individuals with AD, glucose metabolism is reduced, particularly in the temporal and parietal lobes of the brain. These regions are involved in memory and language (Mosconi 2005). Furthermore, the posterior cingulate cortex, a part of the brain involved in memory and attention, often shows decreased glucose metabolism in AD patients (Leech and Sharp 2014). As a result, FDG‐PET has been useful in detecting early signs of neurodegeneration in asymptomatic subjects, and it can aid in the diagnosis and staging of AD (Pérez‐Grijalba et al. 2019). However, in the context of AD, the literature on correlations between miRNA levels and FDG metabolism (FDG‐PET) has been limited. Vergallo et al. conducted a study on sixty individuals with subjective memory complaints and found that miRNA‐15b levels and the hippocampus neuronal metabolism are positively correlated (Vergallo et al. 2021). In our study, we also found that miRNAs are significantly correlated with FDG‐PET, and there is a significant positive correlation between the CSF levels of miR‐210‐3p and miR‐27b‐3p, with glucose hypometabolism in AD patients. While our study primarily highlights significant correlations involving CSF miRNAs, it is important to address the relatively limited findings from plasma samples. The observed lack of significant findings in plasma miRNAs, compared to CSF miRNAs, underscores the inherent challenges of using peripheral biomarkers for neurological diseases. Plasma reflects a mixture of miRNAs derived from various tissues, contributing to systemic noise that can obscure disease‐specific signatures (Cortez et al. 2011). Additionally, the blood‐brain barrier (BBB) restricts the exchange of brain‐derived miRNAs into the circulation, limiting their detectability in plasma (Daneman and Prat 2015). Even when miRNAs cross the BBB, they are subject to degradation, dilution, and potential interference by extracellular vesicles or protein complexes, further complicating their measurement in plasma (Yagi et al. 2017). Despite these challenges, alternative approaches may unlock the diagnostic potential of plasma miRNAs. For instance, extracellular vesicle‐associated miRNAs have shown promise as more robust biomarkers by reducing systemic noise and better reflecting tissue‐specific changes (Gámez‐Valero et al. 2019). Additionally, integrating plasma miRNAs into multi‐modal biomarker panels, which combine data from imaging, protein markers, or metabolomics, may improve sensitivity and specificity (Fiandaca et al. 2014). Furthermore, advanced computational approaches, such as machine learning, have demonstrated the ability to identify complex patterns within plasma miRNA profiles, uncovering their diagnostic potential for diseases like AD (Li et al. 2022). Future studies leveraging these strategies could provide valuable insights into the complementary role of plasma miRNAs alongside CSF‐based biomarkers.

Another modality that is used for the detection of AD is AV45 PET, also known as Florbetapir F 18 PET or Amyloid PET, which is a type of positron emission tomography (PET) imaging technique used to visualize and measure amyloid beta (Aβ) plaques in the brain (Doraiswamy et al. 2012). Aβ plaques are one of the pathological hallmarks of AD, and their accumulation is associated with cognitive decline and neurodegeneration (Villemagne et al. 2013). Previous research has linked miR‐210‐3p to responses to low oxygen levels. It has been found to have increased expression in certain extracellular vesicles from neurons and astrocytes, which could be related to hypoxia. It is suggested that the higher levels of miR‐210‐3p in AD may indicate a hypoxic‐like state in the brain, contributing to the buildup of amyloid‐beta (Kumar et al. 2023). Our study revealed a strong connection between heightened levels of miR‐210‐3p and the buildup of Aβ, aligning with previous research that has identified elevated miR‐210‐3p in the blood of individuals with MCI and AD. These findings suggest that miR‐210‐3p may play a role in the formation of amyloid plaques and could potentially act as an indicator of a hypoxic‐like state in the brain. Moreover, it may be involved in regulating the expression of genes responsible for Aβ metabolism, potentially serving as a vital link between miRNA irregularities and AD development. Given this correlation, it appears that miR‐210‐3p has the potential to serve as both a biomarker for the progression of AD and a promising target for controlling Aβ levels.

Persistent activation of microglia is a finding observed in AD, with heightened concentrations of inflammatory cytokines contributing to this state, which underscores the significant role of neuroinflammation in the progression of neurodegenerative processes (McFarland and Chakrabarty 2023). miR‐223‐3p is a critical regulator of multiple pathways involved in AD pathogenesis, including neuroinflammation, synaptic dysfunction, Aβ metabolism, and neuronal survival. miR‐223‐3p has the capability to shield neurons from deterioration through many pathways (Morquette et al. 2019). One of the pathways is the conversion of M1 microglia into the M2 phenotype (Zhao et al. 2020). This transformation leads to an upregulation of anti‐inflammatory cytokines, a reduction in pro‐inflammatory factor synthesis, an amelioration of neurological impairments, and an augmentation of cognitive functions, including learning and memory capabilities (Zhao et al. 2020). NOD‐like receptor family Pyrin Domain Containing 3 (NLRP3) is another pathway that can be triggered by Aβ, fostering an inflammatory environment in the brain that is implicated in neuronal damage (Guo et al. 2015). miR‐223‐3p is recognized as a negative modulator of NLRP3 expression, evidenced by the observed inverse relationship between the levels of this microRNA and the activation of NLRP3 in mononuclear cells (Guo et al. 2015). Our study revealed a strong connection between miR‐210‐3p levels and Aβ accumulation in the brain. This aligns with previous research that also found an increase in this microRNA in the plasma of individuals with MCI and AD.

## 5 Limitation

Our limitations included a small sample size and a cross‐sectional design, which hinders the capacity to establish a cause‐and‐effect relationship between miRNA levels and the course of AD. The consistency of miRNA detection results may be affected by biological variability and technical limitations, whereas miRNA expression levels can be influenced by potential confounding factors such as comorbid diseases and medication use. Concentrating just on individual miRNAs as biomarkers may not comprehensively capture the intricacies of AD pathogenesis, indicating the necessity for multi‐omics techniques. Furthermore, it is necessary to validate the findings in separate groups of individuals to establish the diagnostic capabilities of the identified miRNAs. Furthermore, the absence of longitudinal data hinders the capacity to monitor alterations in miRNA levels over a period of time and their correlation with the progression of diseases.

## 6 Conclusion

We identified several dysregulated miRNAs, including miR‐30b‐5p, miR‐22‐3p, and miR‐210‐3p, which showed significant associations with AD pathology. Particularly, the  strong positive correlation observed between miR‐210‐3p levels and Aβ burden, and glucose hypometabolism suggests potential role in AD progression. Mechanistically, miR‐210‐3p may contribute to Aβ accumulations by regulating hypoxia‐related pathways and gene expressions involved in amyloid metabolism. Its elevated levels could indicate a hypoxemia‐like state in the AD brain, fostering an environment conducive to Aβ aggregation and neurodegeneration. These findings highlight miR‐210‐3p not only as a biomarker for AD, but also as a potential therapeutic target. While the study primarily highlights correlations involving CSF miRNAs, plasma miRNAs showed fewer significant associations after adjusting for multiple comparisons. This discrepancy may stem from variability introduced by peripheral systemic factors or technical detection limitations. Nevertheless, plasma miRNAs hold promise as accessible and non‐invasive biomarkers, warranting further exploration in larger, longitudinal studies to enhance their diagnostic utility.

## Author Contributions

**Parsa Saberian**: conceptualization, investigation, writing ‐ review and editing, resources, project administration. **Afra Darvishi**: conceptualization, investigation, writing ‐ original draft, writing ‐ review and editing. **Delnia Khezragha**: writing ‐ original draft. **Moojan Forouzandegan**: writing ‐ original draft, writing ‐ review and editing, methodology, project administration. **Mohammad‐Erfan Farhadieh**: software, formal analysis, project administration, data curation, methodology. **Shaghayegh Taghizadeh Khorshidi**: writing ‐ original draft, investigation. **Mohamad Hatami Nejad**: supervision, resources, project administration, writing ‐  and editing. **Sara Sedighi**: writing ‐ original draft, investigation, writing ‐ review and editing. **Reza Barati**: investigation, writing ‐ original draft, writing ‐ review and editing. **Seyed Ahmad Reza Safavi**: writing ‐ original draft, writing ‐ review and editing. **Mohammad Sadeghi**: review and editing, project administration. **David Gulisashvili**: supervision, project administration, writing ‐ review and editing, data curation. **Mahsa Mayeli**: conceptualization, review and editing, supervision. **Shayan Shakeri**: software, visualization.

### Peer Review

The peer review history for this article is available at https://publons.com/publon/10.1002/brb3.70572

## Supporting information



Supporting Information

## Data Availability

Data used in this study were obtained from the Alzheimer’s Disease Neuroimaging Initiative (ADNI) database. Access to the ADNI data is available to qualified researchers upon application at adni.loni.usc.edu. Further information can be obtained from the corresponding author upon reasonable request.
