# Supplementary material for: MicroRNA Signatures Predict Brain Amyloidosis and Neurodegeneration in Alzheimer’s Disease: Insights from [18F] AV45 and FDG PET Imaging
Source: Brain Behav. 2025 May 26;15(5):e70572. doi: 10.1002/brb3.70572 (PMC12105656; doi:10.1002/brb3.70572)

Page 7: Minor typo – change blood serum to blood plasma

Done

Page 8: Add citation to previous miRNA array card protocol

Saugstad JA, Lusardi TA, Van Keuren-Jensen KR, Phillips JI, Lind B, Harrington CA, et al. Analysis of extracellular RNA in cerebrospinal fluid. Journal of extracellular vesicles. 2017;6(1):1317577.

Page 10: Please detail the miRNA QC/normalization that was done in the methods section.

Done, page 7-8.

Page 10: Please add the p-values of the significantly elevated CSF miRNAs.

Done.

How diverse was the study (e.g. # Hispanic, White, Black, etc.)

> Summary (diversity$PTETHCAT)

Unknown Not Hisp/Latino Hisp/Latino

1 150 9

> Summary (diversity$PTRACCAT)

Am Indian/Alaskan Asian

0 3

Hawaiian/Other PI Black

0 6

White More than one

151 0

Unknown

0

What was the distribution of the miRNA concentrations?


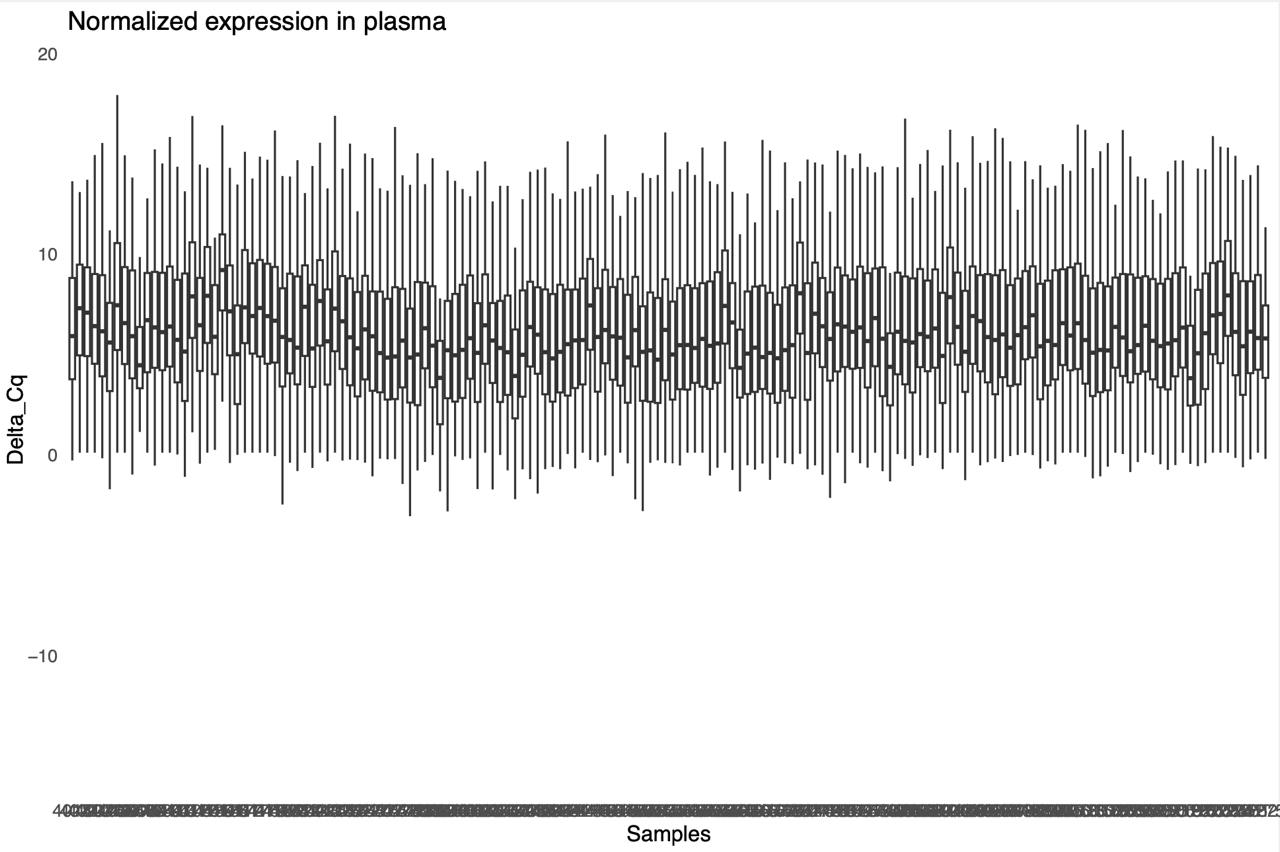

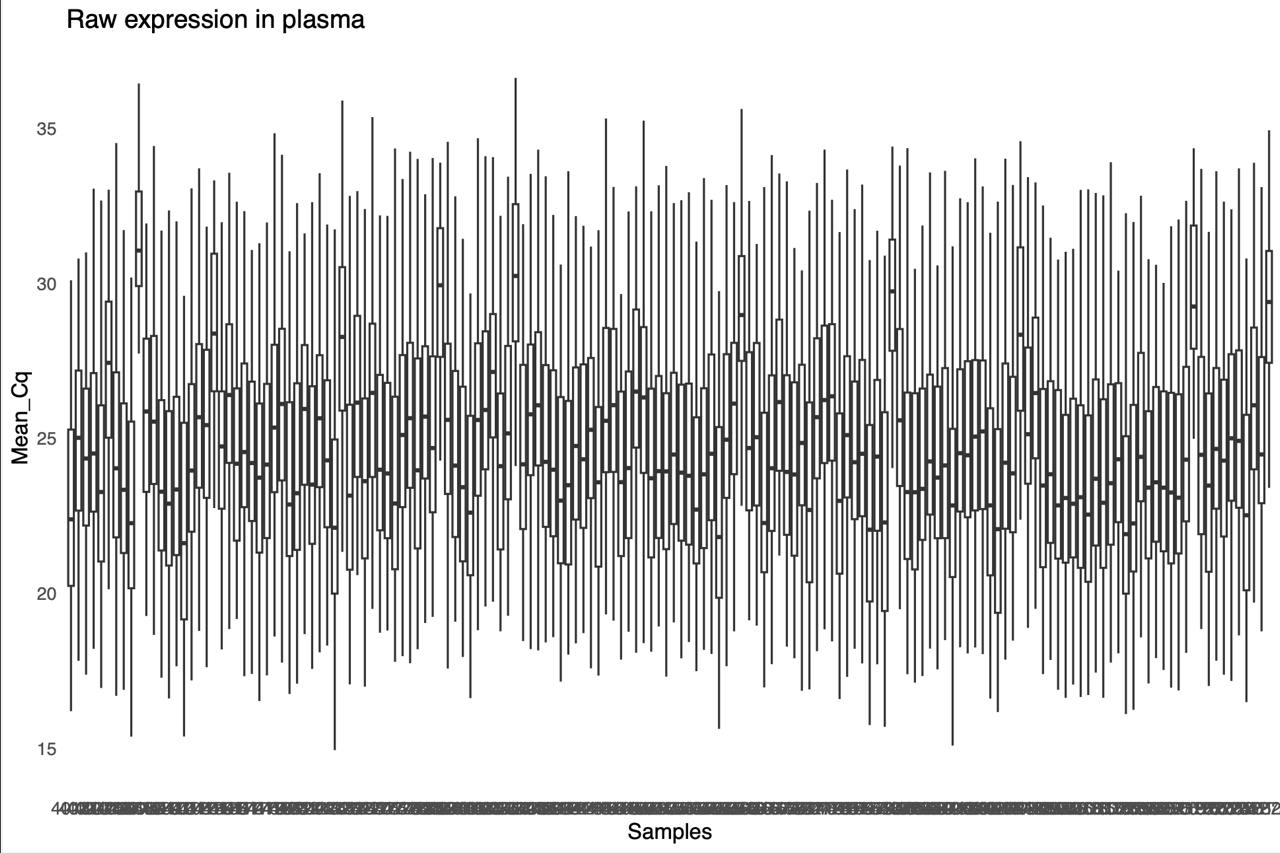

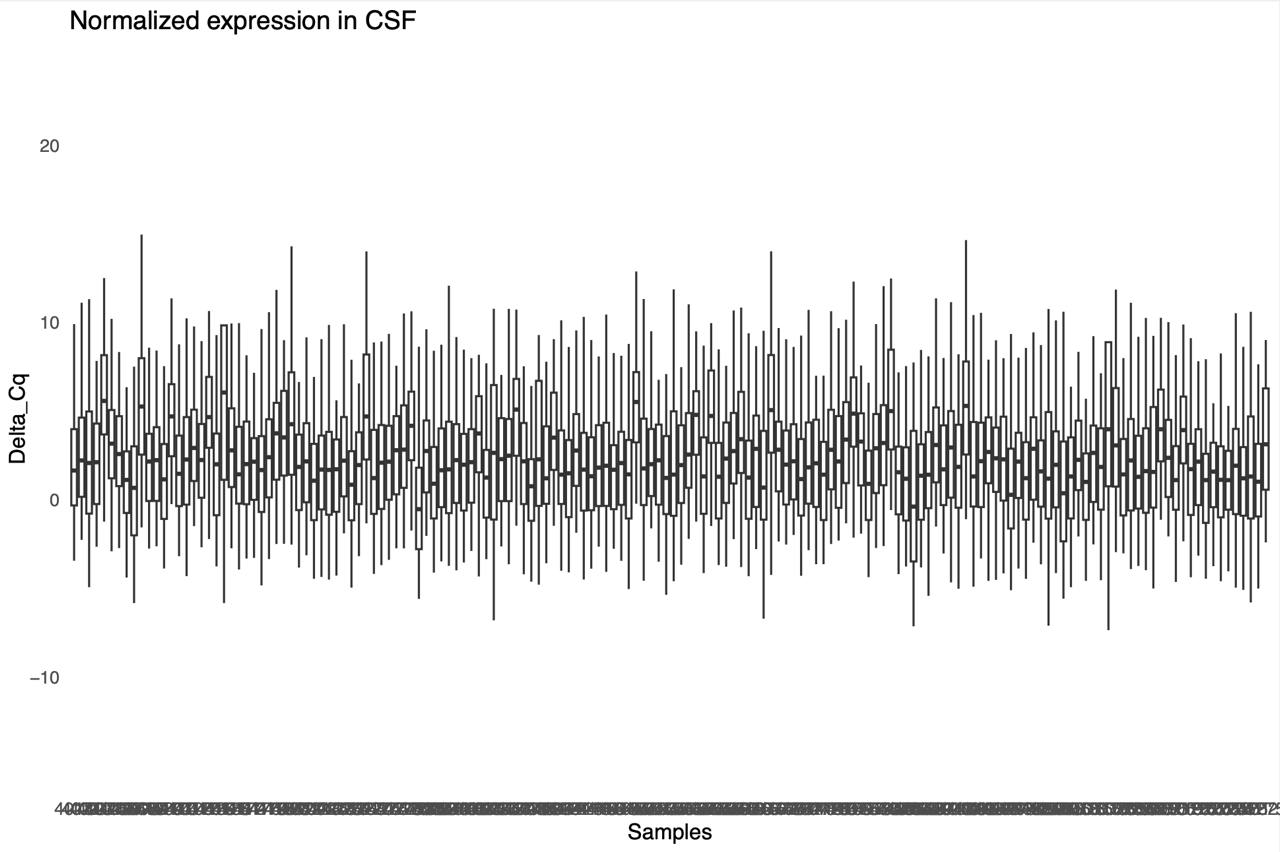

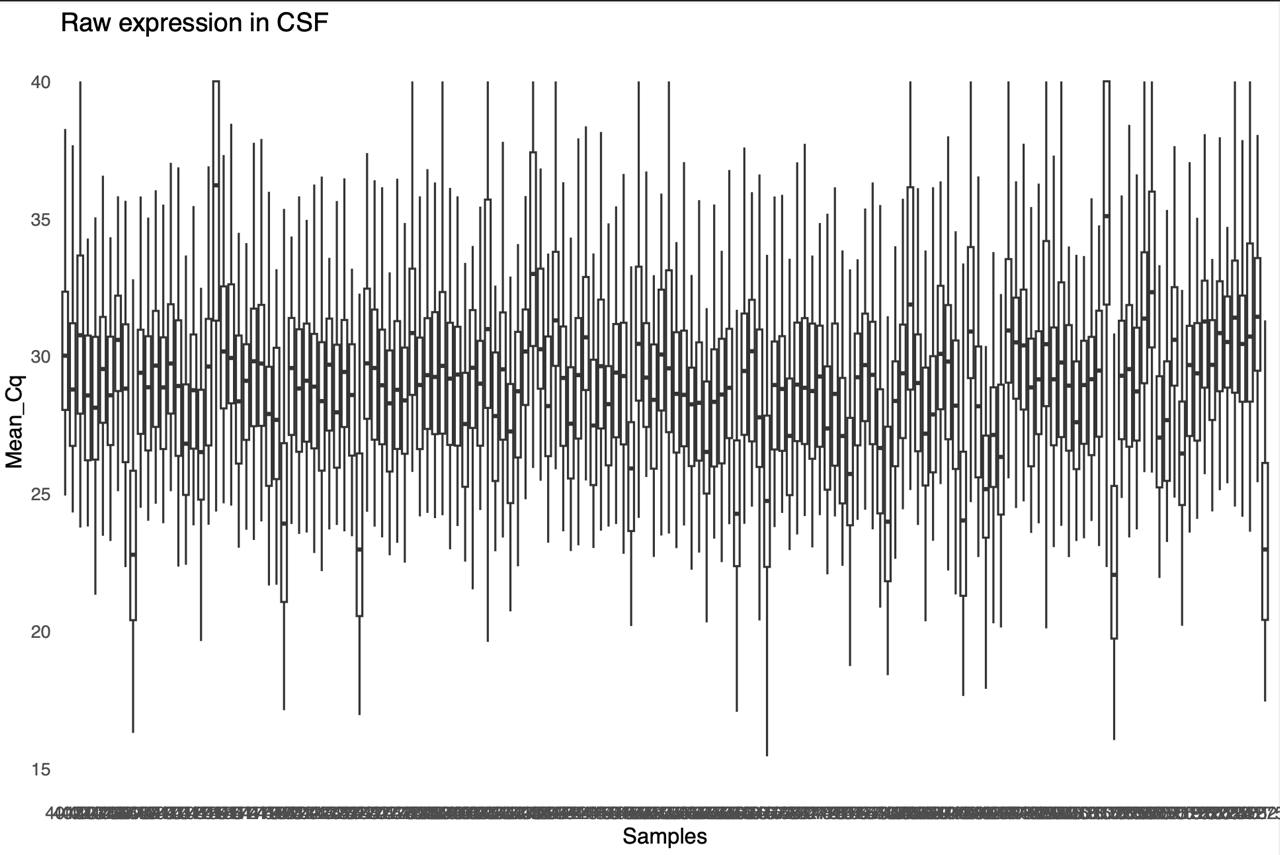

Supplement: Supplementary file 1 — Supporting Information [file BRB3-15-e70572-s001.docx]
